# Structurally-New Hexadecanuclear Ni-Containing Silicotungstate with Catalytic Hydrogen Generation Activity

**DOI:** 10.3390/molecules28052017

**Published:** 2023-02-21

**Authors:** Yequn Wang, Xing Xin, Yeqin Feng, Manzhou Chi, Ruijie Wang, Tianfu Liu, Hongjin Lv

**Affiliations:** MOE Key Laboratory of Cluster Science, Beijing Key Laboratory of Photoelectroic/Electrophotonic Conversion Materials, School of Chemistry and Chemical Engineering, Beijing Institute of Technology, Beijing 102488, China

**Keywords:** polyoxometalates, Ni substitution, photocatalysis, hydrogen production

## Abstract

A structurally-new, carbon-free hexadecanuclear Ni-containing silicotungstate, [Ni_16_(H_2_O)_15_(OH)_9_(PO_4_)_4_(SiW_9_O_34_)_3_]^19-^, has been facilely synthesized using a one-pot, solution-based synthetic method systematically characterized by single-crystal X-ray diffraction and several other techniques. The resulting complex works as a noble-metal-free catalyst for visible-light-driven catalytic generation of hydrogen, by coupling with a [Ir(coumarin)_2_(dtbbpy)][PF_6_] photosensitizer and a triethanolamine (TEOA) sacrificial electron donor. Under minimally optimized conditions, a turnover number (TON) of 842 was achieved for TBA-**Ni_16_P_4_(SiW_9_)_3_**-catalyzed hydrogen evolution system. The structural stability of TBA-**Ni_16_P_4_(SiW_9_)_3_** catalyst under photocatalytic conditions was evaluated by the mercury-poisoning test, FT-IR, and DLS measurements. The photocatalytic mechanism was elucidated by both time-solved luminescence decay and static emission quenching measurements.

## 1. Introduction

Polyoxometalates (POMs), as an important family of metal-oxo nanoclusters with well-defined structures, are typically composed of W, Mo, V, Nb, or Ta ions in their high oxidation states [1,2,3,4]. The research on POM-based transition metal complexes has been attracting increasing attention due to their diverse compositions and versatile physicochemical properties [5,6] in the fields of catalysis [7,8,9,10], medicine [11,12,13,14], molecular magnetism [15,16,17], energy storage/conversion [18,19,20], photochemistry [21,22], etc. Lacunary POMs, with oxygen-rich surfaces and high negative charges [23,24,25], can work as significant multidentate O-donor ligands to coordinate with multiple transition metal ions, leading to the formation of transition-metal-substituted POMs (TMSPOMs). More importantly, the introduction of transition metal ions into POMs could not only adjust the electronic structures of target TMSPOMs, but also introduce more active sites for widespread catalytic applications, especially in photo-driven water splitting area [8,26,27,28]. Given their reversible multi-electron-transfer properties, TMSPOMs usually exhibit the advantages of decent structural stability under the photocatalytic conditions [26,29,30,31]. To date, a series of TMSPOMs have been synthesized and investigated as multi-electron-transfer catalysts for solar energy conversion under visible light irradiation, including water oxidation catalysts (WOCs) [32,33,34,35,36,37,38] and water reduction catalysts (WRCs) [39,40,41,42].

Among these reported TMSPOMs catalysts, a number of multinuclear TMSPOMs have been successfully prepared and systematically characterized. A number of important representative examples of these multinuclear TMSPOMs include {Mn_18_P_8_W_48_} [43], {Mn_19_(SiW_10_)_6_} [44], {Mn_20_P_8_W_48_} [43], {Mn_40_(P_8_W_48_)(P_2_W_14_)_4_(P_2_W_15_)_8_} [45], {Fe_15_(SiW_9_)_4_} [40], {Fe_16_(P_8_W_48_)} [46], {Fe_28_(P_2_W_12_)_4_} [47], {Fe_48_(P_2_W_12_)_8_} [48], {Co_9_(PW_9_)_3_} [49], {Co_16_(PW_9_)_4_} [50], {Co_16_(XW_9_)_4_} (X = Si, As, Ge) [51], {Co_21_(SiW_9_)_6_} [52], {Cu_6_W_18_} [53], {Cu_14_W_36_} [54], and {Cu_20_W_48_} [55]. In addition to these Mn, Fe, Co, Cu-substituted POMs, some multinuclear nickel-containing POMs have also been prepared, including {Ni_12_(SiW_9_)_3_} [56], {Ni_13_(SiW_9_)_3_} [56], {Ni_14_(P_2_W_15_)_4_} [57], {Ni_16_(PW_9_)_4_} [58], {Ni_25_(SiW_9_)_6_} [56], and {Ni_36_(SiW_9_)_6_} [59]. Considering that Ni belongs to the same group as Pt in the periodic table, it could also exhibit catalytic hydrogen evolution activity, even though the activity is much lower than that of Pt [60]. More importantly, the price of Ni is much cheaper with high earth abundance. In nature, Ni has also been successfully used in hydrogenases that can work as an effective hydrogen-evolving catalyst, achieving a turnover frequency (TOF) of 9000 s^−1^ [61,62]. Theoretical studies revealed that the Ni sites in hydrogenases played a crucial role in the hydrogen evolution reaction (HER) [63]. In this context, Ni-substituted POMs will be interesting catalyst candidates for solar-driven hydrogen production, of which, some pioneering works have already been reported [9,58,64,65,66].

Herein, we reported the successful preparation of a structurally-new, carbon-free hexadecanuclear Ni-containing silicotungstate: Na_19_[Ni_16_(H_2_O)_15_(OH)_9_(PO_4_)_4_(A-*α*-SiW_9_ O_34_)_3_]∙57H_2_O (Na-**Ni_16_P_4_(SiW_9_)_3_**), which was systematically characterized by single-crystal X-ray diffraction analysis and other spectroscopic approaches. The resulting tetrabutylammonium (TBA^+^) salt of the polyoxoanion **Ni_16_P_4_(SiW_9_)_3_** (TBA-**Ni_16_P_4_(SiW_9_)_3_**) was investigated as a noble-metal-free catalyst for photo-driven H_2_ production in a typical three-component system.

## 2. Results and Discussion

### 2.1. Structural Description

**Figure 1:**
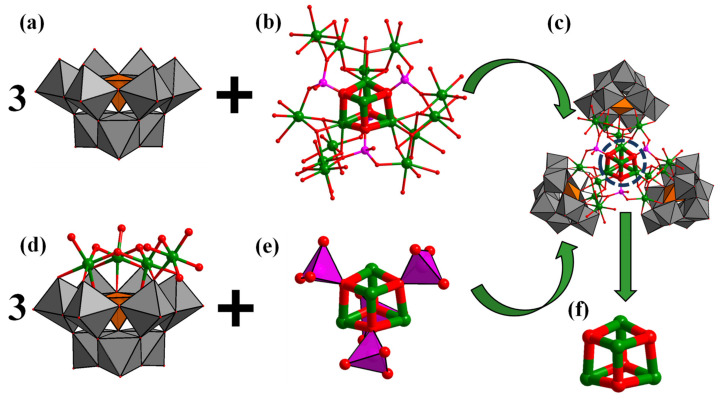
Polyhedral and ball-and-stick representation of the building blocks (**a**,**b**,**d**,**e**) of **Ni_16_P_4_(SiW_9_)_3_**; (**c**) ball-and-stick representation of {Ni_4_O_4_} cubane core; and (**f**) in **Ni_16_P_4_(SiW_9_)_3_**. Color code: WO_6_, grey octahedra; SiO_4_, orange tetrahedra; PO_4_, pink tetrahedra; O, red sphere; Ni, green sphere.

Single crystal X-ray diffraction analysis showed that crystal Na-**Ni_16_P_4_(SiW_9_)_3_** crystallizes in the monoclinic space group C1/c1 (Table 1). The **Ni_16_P_4_(SiW_9_)_3_** polyoxoanion is composed of one central {Ni_16_(H_2_O)_15_(OH)_9_(PO_4_)_3_} ({Ni_16_}) moiety and three lacunary A-*α*-{SiW_9_O_34_} POM ligands (Figure 1a,b). Herein, it is noted that Wang et al. [56] have recently reported a Ni_13_-containing POM cluster. The central {Ni_16_} core is structurally similar to the {Ni_13_} core except that an extra three {Ni(H_2_O)_4_} fragments are incorporated into the middle of the A-*α*-{SiW_9_O_34_} and {PO_4_} group. The central {Ni_16_} core is connected by three μ_3_-{PO_4_} and one μ_4_-{PO_4_} linkers. The whole {Ni_16_} cluster is coordinated by three trivacant A-*α*-{SiW_9_O_34_} POM ligands. In other words, the structure of this polyoxoanion can also be regarded as a central {Ni_4_(H_2_O)_3_(PO_4_)_4_} core stabilized by three {(A-*α*-SiW_9_O_34_)(Ni_4_O(OH)_3_(H_2_O)_4_} units (Figure 1d,e). Such a {Ni_4_(H_2_O)_3_(PO_4_)_4_} core contains a central {Ni_4_O_4_} cubane unit (Figure 1f), which is structurally similar as the Fe_2_S_2_ or FeNiS_2_ clusters working as the catalytically active sites of [FeFe] or [FeNi] hydrogenases, respectively. The Ni−O distances of {Ni_4_O_4_} cubane range from 2.061(16) to 2.175(19) Å and the Ni···Ni distances range from 3.1771(48) to 3.2027(44) Å. The structure of Na-**Ni_16_P_4_(SiW_9_)_3_** exhibits idealized *C_3_* point group symmetry (the *C_3_* axis passing through the corner diagonal of the central {Ni_4_O_4_} cubane). All the nickel ions are in the +2 oxidation states as confirmed by the BVS calculations.

**Figure 2:**
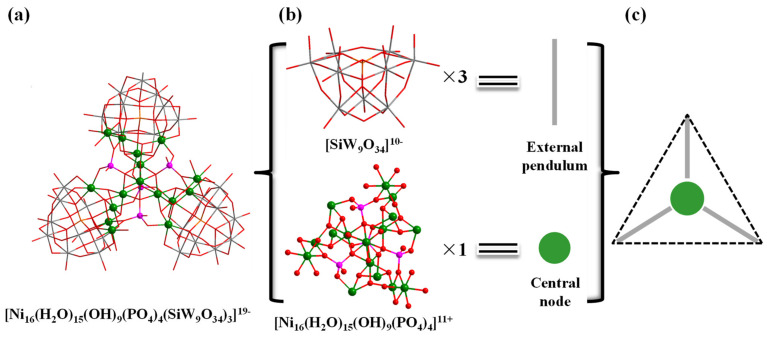
Combined wireframe/ball-and-stick representation of **Ni_16_P_4_(SiW_9_)_3_**: (**a**) **[Ni_16_(H_2_O)_15_(OH)_9_(PO_4_)_4_(SiW_9_O_34_)_3_]^19-^**; (**b**) Representations of the building blocks of **Ni_16_P_4_(SiW_9_)_3_**; and (**c**) Triangular geometry of **Ni_16_P_4_(SiW_9_)_3_**. Color code: W grey, Ni green, P pink, Si orange, O red.

In addition, the structure of polyoxoanion **Ni_16_P_4_(SiW_9_)_3_** (Figure 2a) can also be simplified into a ball-and-stick model, therein the {SiW_9_O_34_} moieties and the central {Ni_16_} unit are regarded as external pendulum and central node (Figure 2b), respectively. Three external pendulums and one central node can be assembled into a triangular geometry (Figure 2c). Interestingly, it is noted that the molecular structural units of polyoxoanion **Ni_16_P_4_(SiW_9_)_3_** can form a zigzag one-dimensional (1-D) chain connected by Na^+^ counter cations along *a* axis (Figure S1).

### 2.2. Characterization of Ni_16_P_4_(SiW_9_)_3_

The FT-IR spectrum of Na-**Ni_16_P_4_(SiW_9_)_3_** was collected in a 2 wt% KBr pellet in the region of 4000 to 400 cm^−1^ (Figure S2, black curve). The signal at 987 cm^−1^ are attributed to vibrations of W-O_t_. The W-O-W vibrations peaks are located at 889, 861, 810, and 684 cm^−1^, while the absorption peak at 937 cm^−1^ is consistent with Si–O_a_ vibrations. All the characteristic bands of the Na-**Ni_16_P_4_(SiW_9_)_3_** structure were observed in the FT-IR spectrum, which is similar to that of the lacunary [A-*α*-SiW_9_O_34_]^10−^ POM ligand (Figure S2, blue curve). The replacement of Na^+^ cations with tetrabutylammonium (TBA^+^) retains the molecular skeleton of **Ni_16_P_4_(SiW_9_)_3_** (Figure S2, red curve), the corresponding vibrational signals of TBA^+^ cation is well observed [67,68]. The UV-vis spectrum of polyoxoanion Na-**Ni_16_P_4_(SiW_9_)_3_** exhibits a strong absorption peak in the UV region, which can be assigned as the oxygen-to-metal charge-transfer that are typically observed in the POM structures (Figure S3). Thermogravimetric analysis (TGA) showed a weight loss of 10.5%, which was calculated to be 57 crystallization H_2_O molecules in one formula unit (Figure S4). Due to incomplete substitution of Na^+^ in the crystal, the amount of TBA^+^ was determined by the TGA. The TGA of TBA-**Ni_16_P_4_(SiW_9_)_3_** shows ~2.97% weight loss ~at 20–100 ℃ and 27.5% weight loss at 100–750 ℃, roughly corresponding to about 15 H_2_O molecules and 14 TBA^+^ cations to replace the initial Na^+^ cations, respectively. The chemical compositions of polyoxoanion Na-**Ni_16_P_4_(SiW_9_)_3_** were characterized by ICP-AES tests (see Experimental section). Then, XPS data was further collected to characterize the existence and oxidation states of Ni (Figure S5a), P (Figure S5b), Si (Figure S5c), and W (Figure S5d) elements in complex Na-**Ni_16_P_4_(SiW_9_)_3_** (Figure S5). For instance, the binding energies of the Ni 2p_3/2_ and 2p_1/2_ (with corresponding satellite peaks at 862.5 eV and 880.3 eV, Figure S5a) peaks were located at 856.0 and 874.0 eV (Figure S5), respectively, indicating the +2 oxidation state of the Ni centers in the cluster. The XPS results are in good consistence with the BVS calculations. In addition, SEM/EDX results also revealed the microscopic morphology of the Na-**Ni_16_P_4_(SiW_9_)_3_** crystal and the existence of Si, Ni, P, and W elements (Figure S6). The calculated atomic ratio of Ni/W (1:1.50) from the EDX results is in good agreement with the theoretical value (1:1.69) (Figure S7). In addition, the PXRD pattern of Na-**Ni_16_P_4_(SiW_9_)_3_** matched well with the simulated diffraction pattern, also indicating the phase purity of the title compound (Figure S8).

### 2.3. HOMO and LUMO Investigation

Electrochemical measurements and UV–vis absorption spectrum were conducted to calculate the HOMO and LUMO energy levels of the catalyst. Estimated from the UV–vis absorption spectrum, the energy bandgap of the crystal was obtained, thus the HOMO was calculated to be the sum of the energy bandgap and LUMO (Figure S9). The LUMO orbital energy for the TBA-**Ni_16_P_4_(SiW_9_)_3_** was measured as −3.31 eV. According to the UV-vis-NIR spectra of K-M function vs. energy (eV), the energy gap was 2.40 eV. Therefore, the HOMO orbital energy was −5.71 eV. In the previous research of our group, the orbital energy of photosensitizer [Ir(coumarin)_2_(dtbbpy)]^+^ was calculated. Its LUMO and HOMO energies were −3.28 and −5.42 eV, respectively [69]. Therefore, the LUMO electron of excited state [Ir(coumarin)_2_(dtbbpy)]^+^* can be transferred to the LUMO orbitals of the catalyst, providing the possibility for establishing a photocatalytic hydrogen production system.

### 2.4. Photocatalytic Hydrogen Production and Evaluation of Catalyst Stability

**Figure 3:**
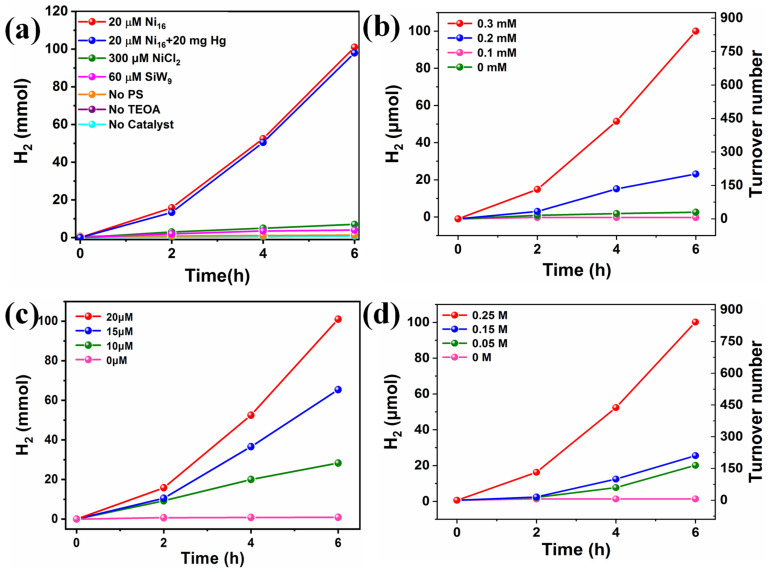
(**a**) photocatalytic H_2_ evolution using different catalysts and control experiments and varying concentrations of, (**b**) [Ir(coumarin)_2_(dtbbpy)]^+^ photosensitizer, (**c**) TBA-**Ni_16_P_4_(SiW_9_)_3_** catalyst, and (**d**) TEOA sacrificial agent. Standard reaction conditions: 300 W Xe lamp with a 400 nm cutoff filter, [Ir(coumarin)_2_(dtbbpy)]^+^ (0.3 mM), TEOA (0.25 M), H_2_O (2 M), catalyst (20 μM), CH_3_CN/DMF (*v*/*v* = 1/3) deaerated with Ar/CH_4_ (*v*/*v* = 4/1).

Considering the vital challenges of energy shortage and environmental problems faced by modern mankind, the development of clean and renewable energy alternatives has been attracting tremendous research attention. Photocatalytic hydrogen production driven by solar energy represents a promising way to produce clean secondary energy carriers. Herein, the visible light-driven H_2_ production activity of TBA-**Ni_16_P_4_(SiW_9_)_3_** was investigated in a well-established three-component system by using [Ir(coumarin)_2_(dtbbpy)]^+^ [69] as the photosensitizer, TBA-**Ni_16_P_4_(SiW_9_)_3_** as the WRC, and TEOA as electron donor in a mixed CH_3_CN/DMF (*v*/*v* = 1/3) solvent. The reaction solution was exposed to 400 nm visible-light irradiation at room temperature. All turnover numbers (TONs) were calculated with respect to TBA-**Ni_16_P_4_(SiW_9_)_3_** catalyst. The effect of each component on photocatalytic activity was evaluated by different control experiments. As shown in Figure 3a, the catalytic system in the absence of photosensitizer, sacrificial reagent, or catalyst produces negligible H_2_ production under otherwise identical conditions. In addition, the replacement of the catalyst TBA-**Ni_16_P_4_(SiW_9_)_3_** with the lacunary {**SiW_9_**} POM stabilizing ligand causes very low amounts of H_2_ production, proving the vital role of Ni sites. Moreover, the catalytic system using stoichiometric equivalents of NiCl_2_ (320 μM) as that of 20 μM TBA-**Ni_16_P_4_(SiW_9_)_3_** shows a remarkable decrease in hydrogen production. These results demonstrate that photosensitizer, sacrificial agent, and catalyst are all necessary component in the photocatalytic process. More importantly, the structural skeleton of TBA-**Ni_16_P_4_(SiW_9_)_3_** is essential for efficient catalysis because the unique molecular structure of TBA-**Ni_16_P_4_(SiW_9_)_3_** polyoxoanion can work as electron reservoir to effectively store electrons in the electron-deficient POM ligands and, in the meantime, supply electrons to the catalytically active Ni centers, thereby leading to the high catalytic efficiency of TBA-**Ni_16_P_4_(SiW_9_)_3_**. The different concentrations of each component also significantly affect H_2_ production (Figure 3b–d). Increasing the concentration of [Ir(coumarin)_2_(dtbbpy)]^+^ photosensitizer from 0.1 to 0.3 mM enhances the H_2_ yield from ~3.6 to ~100 μmol, corresponding to a TON change from ~30 to ~842 (Figure 3b). The catalytic performance of this TBA-**Ni_16_P_4_(SiW_9_)_3_** catalyst is comparable to that of some known Ni-containing POMs under homogeneous catalytic systems using Ir/Ru-based photosensitizers (Table S3). While adjusting the concentration of TEOA from 0.05 M to 0.25 M, the H_2_ production increases from ~20 to ~100 μmol. In addition, the H_2_ yield was enhanced from ~0.9 to ~164 μmol as the concentration of TBA-**Ni_16_P_4_(SiW_9_)_3_** changed from 5 to 20 μM. Based on the above experimental results, the optimal combination of the [Ir(coumarin)_2_(dtbbpy)]^+^ photosensitizer, TEOA electron donor, and TBA-**Ni_16_P_4_(SiW_9_)_3_** catalyst are vital for highly efficient photocatalytic H_2_ evolution.

The stability of catalyst agent has been a general concern in molecular photocatalytic systems. In this paper, the stability of TBA-**Ni_16_P_4_(SiW_9_)_3_** was assessed using a range of optical methods and experimental evaluation. To investigate whether the TBA-**Ni_16_P_4_(SiW_9_)_3_** catalyst was decomposed into the Ni nanoparticles, we have carried out a mercury-poisoning test by the addition of 20 mg Hg to the photocatalytic solution. The addition of Hg does not significantly affect the hydrogen production, implying the integrity of TBA-**Ni_16_P_4_(SiW_9_)_3_** catalyst during photocatalysis (Figure 3a). Moreover, to further characterize the stability of TBA-**Ni_16_P_4_(SiW_9_)_3_** catalyst, the post-reaction catalyst was isolated in the form of [Ru(bpy)_3_]_x_-**Ni_16_P_4_(SiW_9_)_3_** adducts after photocatalysis by adding cationic [Ru(bpy)_3_]^2+^ species. FT-IR spectra of isolated [Ru(bpy)_3_]_x_-**Ni_16_P_4_(SiW_9_)_3_** adducts reveal almost no changes before and after photocatalysis for 6 h (Figure S10), implying the decent molecular stability of the TBA-**Ni_16_P_4_(SiW_9_)_3_** catalyst. The DLS measurement illustrated a signal centered at ~1.7 nm for the TBA-**Ni_16_P_4_(SiW_9_)_3_** (20 μM) system after 6 h of catalysis (Figure S11), which is consistent with the size of TBA-**Ni_16_P_4_(SiW_9_)_3_**, about 18.98 Å, implying the integrity of the TBA-**Ni_16_P_4_(SiW_9_)_3_** polyoxoanion.

### 2.5. Photocatalytic Mechanistic Studies

**Figure 4:**
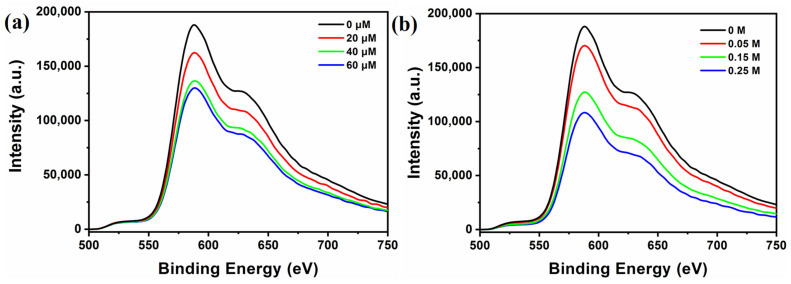
Emission quenching of excited state [Ir(coumarin)_2_(dtbbpy)]^+^* (0.3 mM) by: (**a**) TBA-**Ni_16_P_4_(SiW_9_)_3_**, and (**b**) TEOA in 3 mL of CH_3_CN/DMF (*v*/*v* = 1/3) solution upon 450 nm excitation.

It is known that the photoexcited photosensitizer can work as both oxidizing and reducing species in the typical photocatalytic systems. Therefore, to reveal the photocatalytic mechanism, the quenching experiments of [Ir(coumarin)_2_(dtbbpy)]^+^ by TEOA and TBA-**Ni_16_P_4_(SiW_9_)_3_** has been performed in CH_3_CN/DMF using both steady-state emission quenching and time-resolved luminescence decay spectroscopy. As shown in Figure 4, a strong emission band in the region of 500−750 nm was observed upon excitation of [Ir(coumarin)_2_(dtbbpy)]^+^ (λ_e_ = 460 nm), and the emission intensity of [Ir(coumarin)_2_(dtbbpy)]^+^ was progressively quenched with the addition of TBA-**Ni_16_P_4_(SiW_9_)_3_** (0–60 μM) and TEOA (0–0.25 M). Luminescence quenching rate constants can be derived by the Stern–Volmer plot using a linear function (Figure S12). The quenching rate constant (k_rq_) for the reductive pathway by TEOA was calculated as 2.55 × 10^6^ M^−1^·s^−1^, while the oxidative quenching rate constant (k_oq_) by TBA-**Ni_16_P_4_(SiW_9_)_3_** was 6.27 × 10^9^ M^−1^·s^−1^. It is clear that the k_oq_ value is three orders of magnitude higher than the k_rq_ value, which can be attributed to the strong electrostatic interaction between positively-charged [Ir(coumarin)_2_(dtbbpy)]^+^ photosensitizer and negatively-charged TBA-**Ni_16_P_4_(SiW_9_)_3_** catalyst. However, in the typical photocatalytic hydrogen evolution experiments, the concentrations of TEOA and TBA-**Ni_16_P_4_(SiW_9_)_3_** were 0.25 M and 20 μM, respectively. Therefore, the corresponding quenching rates can be calculated by multiplying the values of quenching rate constants by the concentrations of quenchers, leading to the values of 0.6375 × 10^6^ s^−1^ by TEOA and 1.254 × 10^5^ s^−1^ by TBA-**Ni_16_P_4_(SiW_9_)_3_** catalyst. Such relatively higher quenching rates by TEOA revealed that that the reductive pathway was still the dominant one during photocatalysis (Figure S13).

**Figure 5:**
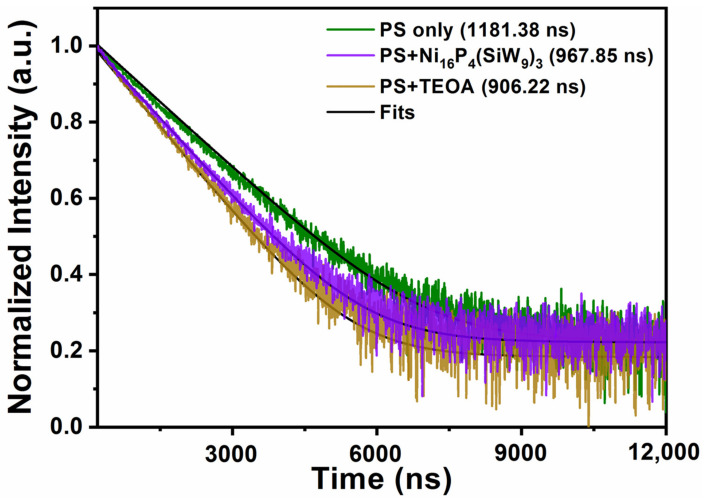
Normalized luminescence decay kinetics of [Ir(coumarin)_2_(dtbbpy)]^+^ (green curve) by TBA-**Ni_16_P_4_(SiW_9_)_3_** (purple curve) and TEOA (brown curve). Conditions: 460 nm excitation, 0.3 mM [Ir(coumarin)_2_(dtbbpy)]^+^, 0.25 M TEOA or 20 μM catalyst, CH_3_CN/DMF (*v*/*v* = 1/3). The black curves are the single-exponential fitting results.

By using the time-resolved fluorescence spectroscopy, the decay kinetics of the excited state [Ir(coumarin)_2_(dtbbpy)]^+^* was also investigated. The experimental phenomenon that both TBA-**Ni_16_P_4_(SiW_9_)_3_** and TEOA can accelerate the decay of [Ir(coumarin)_2_(dtbbpy)]^+^* luminescence was obvious (Figure 5). The single-exponential fitting of decay kinetics of [Ir(coumarin)_2_(dtbbpy)]^+^* yielded a lifetime of ~1181.38 ns, which was further decreased to ~906.22 and ~967.85 ns in the presence of TEOA and TBA-**Ni_16_P_4_(SiW_9_)_3_**, respectively. These results clearly revealed that both reductive and oxidative quenching processes existed during photocatalysis and the reductive quenching process is the dominant one, thus agreeing with the steady-state emission quenching results.

According to the above mechanistic analyses, the possible photocatalytic process was proposed as follows. Under the light irradiation, the photons were absorbed by the [Ir(coumarin)_2_(dtbbpy)]^+^ photosensitizer, generating the excited state [Ir(coumarin)_2_(dtbbpy)]^+^*. In addition to the oxidative quenching of [Ir(coumarin)_2_(dtbbpy)]^+^* by TBA-**Ni_16_P_4_(SiW_9_)_3_** catalyst, the photoexcited states can also be reductively quenched by TEOA to form one-electron-reduced [Ir(coumarin)_2_(dtbbpy)] species. The TBA-**Ni_16_P_4_(SiW_9_)_3_** catalyst can be reduced by accepting electrons from this reduced [Ir(coumarin)_2_(dtbbpy)] species. During photocatalysis, the lacunary {SiW_9_} POM building blocks and transition metals act as electron storage mediator and catalytic active sites, respectively. The lacunary {SiW_9_} POM ligands can be reduced by reversibly storing multiple electrons and protons, then the electrons could be continuously utilized by the Ni active centers to effectively catalyze hydrogen evolution.

## 3. Experimental Section

### 3.1. Methods and Materials

All chemicals were used as received without further purification, unless otherwise specified. Trivacant lacunary POM Na_10_[A-α-SiW_9_O_34_]·18H_2_O was synthesized according to the literature method [70]. Single-crystal X-ray crystallography was performed on a Bruker APEXII DUO (Bruker, Karlsruhe, Germany) diffractometer CCD detector operated at 40 kV and 40 mA with Mo Kα radiation (λ = 0.71073 Å). Fourier transform infrared (FT-IR) were recorded on a Bruker TENSOR II spectrometer (Bruker, Karlsruhe, Germany) with ~2 wt% KBr pellets. Ultraviolet-visible (UV-Vis) absorption spectra were measured by using a Techcomp UV 2600 (Techcomp, Shanghai, China) spectrophotometer. Scanning electron microscopy (SEM) associated with energy-dispersive X-ray spectroscopy (EDX) data were collected on a JSM-7500F (JEOL, Tokyo, Japan) instrument. ICP-AES was conducted on an Agilent ICP-AES 5110 (Agilent, Santa Clara, CA, USA) to analyze the elemental composition of the resulting complex, which contains Ni, Si, P, W, and Na. Thermogravimetric data (TGA) were collected on a HITACHI TG/DTA7300 (HITACHI High-Technologies, Yamaguchi, Japan) instrument from 20 to 800 °C under N_2_ atmosphere. X-ray photoelectron spectroscopy (XPS) measurements were performed on a PHI 5000 Versaprobe III (Ulvac-Phi, Osaka, Japan) instrument. The X-ray powder diffraction pattern was collected on a Shimadzu XRD-6000 instrument (Shimadzu, Kyoto, Japan).

### 3.2. Synthesis of Na_19_[Ni_16_(H_2_O)_15_(OH)_9_(PO_4_)_4_(A-α-SiW_9_O_34_)_3_]∙57H_2_O (Na-**Ni_16_P_4_(SiW_9_)_3_**)

NiCl_2_·6H_2_O (0.15 g, 0.32 mmol) was dissolved in 10 mL distilled water. Then, Na_10_[A-*α*-SiW_9_O_34_]·18H_2_O (0.25 g, 0.08 mmol) was added with stirring until a clear and green solution was obtained. The pH value of the resulting mixture was adjusted to 7.5 with 4.0 M NaOH solution. The turbid solution was stirred for 30 min at room temperature. Then, solid Na_3_PO_4_·12H_2_O (0.57 g, 1.5 mmol) was added, while maintaining above pH at 7.5 with 2.0 M HCl. The admixture was heated to 80 °C for 2 h and the reaction solution became clear during the heating process. Then, the light green precipitate was removed by filtration. The clear filtrate was kept in a 25 mL beaker to allow slow evaporation at room temperature. After about 5 days, the desired rhombohedral flake green crystals were obtained by filtration and further dried at ambient conditions. Yield: 83 mg (40.5% based on Ni). Analytical calculated (found, wt%) compositions of complex Na-**Ni_16_P_4_(SiW_9_)_3_**: Na 4.42 (4.15), Si 0.85 (0.90), P 1.25 (1.23), Ni 9.50 (9.06), W 50.20 (49.59).

The TBA^+^ salt of **Ni_16_P_4_(SiW_9_)_3_** (TBA**-Ni_16_P_4_(SiW_9_)_3_**) was synthesized using the following procedure: the crystalline Na**-Ni_16_P_4_(SiW_9_)_3_** (0.2 g, 17.86 μmol) sample was dissolved in 5 mL of H_2_O, to which a solution of TBA bromide (5 g, 15.6 mmol) in 5 mL of 0.5 M sodium acetate buffer (pH 4.8) was added and then stirred vigorously for 0.5 h. The light green precipitate was formed, separated by centrifugation, washed with ice water to remove excess TBA bromide, and finally dried under vacuum. The final TBA-**Ni_16_P_4_(SiW_9_)_3_** product was collected and characterized by FT-IR and TGA. The empirical molecular formula of the TBA^+^ salt of **Ni_16_P_4_(SiW_9_)_3_** was calculated as TBA_14_Na_5_[Ni_16_(H_2_O)_15_(OH)_9_(PO_4_)_4_(A-α-SiW_9_O_34_)_3_]∙15H_2_O.

### 3.3. Single-Crystal X-Ray Crystallography

An appropriate high-quality crystal (0.19 × 0.20 × 0.21 mm^3^) was selected and encapsulated in a single crystal tube with Vaseline on both ends for data collection at 298 K. The data of Na-**Ni_16_P_4_(SiW_9_)_3_** crystal was collected on a Bruker APEXII diffractometer. The APEX 3 software (APEX3 v2016.1-0, Bruker, Karlsruhe, Germany) was installed on the diffractometer for data collection, indexing, and initial cell refinements [71]. Optimal reflections have been collected for high-quality frame integration and final cell refinements using SAINT software (APEX3 v2016.1-0, Bruker, Karlsruhe, Germany) [72]. The Olex 2 software (Olex2 v1.3, England) equipped with Superflip structure solution program was used to solve the crystal structures, which were further refined by least squares using ShelXL [73,74,75]. All these non-H atoms were refined with anisotropic thermal parameters. The hydrogen atoms were located by bond valence sum (BVS) calculations. Details of the crystallographic data and analyses for the compound Na-**Ni_16_P_4_(SiW_9_)_3_** are given in Table 1, and important bond lengths as well as corresponding bond valence sum (BVS) calculations are summarized in Tables S1 and S2. The Cambridge Crystallographic Data Centre (CCDC) number of Na-**Ni_16_P_4_(SiW_9_)_3_** is deposited as 2223896. Further details on the crystal structure investigations may be obtained from http://www.ccdc.cam.ac.uk/deposit (accessed on 12 February 2022) on quoting the depository number as mentioned.

### 3.4. Photocatalytic Hydrogen Evolution Tests

Photocatalytic hydrogen production was conducted in degassed CH_3_CN-DMF (*v*/*v* = 1/3) solution containing triethanolamine (TEOA) electron donor, H_2_O proton source, iridium complexes ([Ir(coumarin)_2_(dtbbpy)][PF_6_]) photosensitizers [69], and Ni-substituted polyoxometalate TBA-**Ni_16_P_4_(SiW_9_)_3_** as the catalyst. The reaction solution was deaerated with Ar/CH_4_ (*v*/*v* = 4/1), the internal standard of CH_4_ was used for better quantification. The degassed mixture was illuminated using a 300 W Xe-lamp (PerfectLight, Beijing, China) equipped with a 400 nm cutoff filter at room temperature with constant stirring. Gas chromatograph (Thermo GC7900, thermal conductivity detector (TCD), Thermo Fisher Scientific, Waltham, MA, USA) was used to analyze the hydrogen in the reaction headspace using a 5 Å molecular sieve capillary column. All turnover numbers (TONs) were calculated based on catalyst TBA-**Ni_16_P_4_(SiW_9_)_3_**. Control experiments were conducted under similar experimental conditions by removing one component one at a time. Additional control experiments were conducted by replacing the TBA-**Ni_16_P_4_(SiW_9_)_3_** catalyst with NiCl_2_ or trivacant {**SiW_9_**} POM under otherwise identical conditions.

### 3.5. Electrochemical Measurements

The cyclic voltammetry was conducted on a CHI660E (Chinstruments, Shanghai, China) instrument using 0.1 M tetrabutylammonium hexafluorophosphate (TBAPF_6_) as electrolyte, glassy carbon as working electrode, Pt wire as counter electrode, and non-aqueous Ag/Ag^+^ as reference electrode. The scan rate of CV was 50 mV/s. The E_ox_ or E_red_ was measured by using the internal standard substance ferrocene (E_ox_(Fc/Fc+) = 0 V vs. Ag/Ag+) [76]. The working electrode was treated by grinding with 0.3 μm and 0.05 μm alumina for about 4 min, flushing with deionized water, sonicating with acetone for 2 min and drying with nitrogen gas flow. The solution was degassed to remove oxygen. At the time of measurement, the degassing device is placed above the liquid level to avoid large disturbance. The HOMO and LUMO energy levels was calculated by the following equations:

HOMO = −[E_ox_ − E(Fc/Fc+) + 4.8] eV (1)

LUMO = −[E_red_ − E(Fc/Fc+) + 4.8] eV (2)

### 3.6. Steady-State and Time-Resolved Fluorescence Decay Measurements

The Edinburgh Instruments FS5 (Edinburgh Instruments, Livingston, UK) spectrofluorometer was used to test the steady-state luminescence quenching spectra and time-resolved luminescence decay kinetics. The solvent for photoluminescence decay measurements was mixing CH_3_CN/DMF with a volume ratio of 1:3. The different concentrations of TBA-**Ni_16_P_4_(SiW_9_)_3_** or TEOA were degassed with Ar for 10 min to avoid the influence of oxygen before experiment. An intense emission band of [Ir(coumarin)_2_(dtbbpy)]^+^ at 450–750 nm (λ_excitation_ = 450 nm) was recorded and an EPL-450 picosecond pulsed diode laser system (pulse output 450 nm) was used to measure the emission lifetime at the emission maximum.

## 4. Conclusions

In summary, we reported the successful synthesis of a structurally-new, carbon-free hexadecanuclear Ni-containing silicotungstate, [Ni_16_(H_2_O)_15_(OH)_9_(PO_4_)_4_(SiW_9_O_34_)_3_]^19-^, using a one-pot, solution-based synthetic method. A series of techniques, including single-crystal X-ray diffraction, UV-Vis, TGA, FT-IR, SEM/EDS, XPS, etc., have systematically characterized the chemical composition, crystal structure, and elemental oxidation states of the resulting POM complex. While coupling with a [Ir(coumarin)_2_(dtbbpy)][PF_6_] photosensitizer and a triethanolamine (TEOA) sacrificial electron donor, the title TBA-**Ni_16_P_4_(SiW_9_)_3_** polyoxoanion works as a noble-metal-free catalyst for hydrogen generation under visible light irradiation, achieving a turnover number (TON) of 842 under minimally optimized conditions. The mercury-poisoning test, FT-IR spectra of the isolated [Ru(bpy)_3_]_x_-**Ni_16_P_4_(SiW_9_)_3_** adducts, and the DLS measurements revealed the structural stability of TBA-**Ni_16_P_4_(SiW_9_)_3_** catalyst under photocatalytic conditions. More importantly, both time-solved luminescence decay and static emission quenching measurements elucidated the photocatalytic mechanism, confirming the existence of both reductive and oxidative quenching pathways with the reductive quenching pathway as the dominant one. This work presents another good example of using Ni-substituted POMs as efficient hydrogen evolution catalyst, which might provide insights for future design of additional high-nuclearity metal-containing POMs as catalysts for solar energy conversion.

## Figures and Tables

**Table 1:** Crystal data and structure refinements for **Ni_16_P_4_(SiW_9_)_3_**.

Compound	Ni_16_P_4_(SiW_9_)_3_
Empirical formula	Na_19_H_153_Ni_16_P_4_Si_3_W_27_ O_199_
*M*_r_ (g mol^−1^)	9885.82
Temperature/K	298(2)
Crystal system	Monoclinic
Space group	*C1/c1*
*a* (Å)	37.2051(18)
*b* (Å)	18.3718(9)
*c* (Å)	26.9158(13)
*α* (°)	90.00
*β* (°)	101.907(2)
*γ* (°)	90.00
*V*/Å^3^	18,001.8(15)
*Z*	4
*ρ*_calcd_ (g cm^−3^)	3.127
μ mm^−1^	18.953
*F*(000)	14,824
Limiting indices	−44 ≤ h ≤ 44, −21 ≤ k ≤ 21, −32 ≤ l ≤ 32
Reflections collected	97,803
Independent reflections	37,240
Restrains/parameters	2/1238
*θ*_min_/*θ*_max_	1.241/24.999
*R*_int_	0.0848
GooF	0.960
*R* [*I* > 2*σ*]	*R*_1_ = 0.0402, w*R*_2_ = 0.0666
*R*(all data)	*R*_1_ = 0.0687, w*R*_2_ = 0.0752
***R*_1_ = ∑(|*F*_o_| − |*F*_c_||/∑|*F*_o_|, w*R*_2_ = {∑[w(*F*^2^_o_ − *F*^2^_c_)^2^]/∑[w(*F*^2^_o_)^2^]}^1/2^.**	

## Data Availability

Not applicable.

## Supplementary Materials

The following supporting information can be downloaded at: https://www.mdpi.com/article/10.3390/molecules28052017/s1, Tables S1–S3: BVS calculations, and a comparison of Ni-POMs HER; Figures S1–S13: crystal packing, FT-IR, UV-Vis, TGA, CV, XPS, SEM/EDX, DLS, Stern–Volmer plots, and Emission quenching spectra [10,58,64,65,77,78,79,80].

## Sample Availability

Samples of the compounds are available from the authors.

## References

[1] Huang P., Qin C., Su Z.-M., Xing Y., Wang X.-L., Shao K.-Z., Lan Y.-Q., Wang E.-B. (2012). Self-Assembly and Photocatalytic Properties of Polyoxoniobates: {Nb_24_O_72_}, {Nb_32_O_96_}, and {K_12_Nb_96_O_288_} Clusters. J. Am. Chem. Soc..

[2] Lydon C., Sabi M.M., Symes M.D., Long D.-L., Murrie M., Yoshii S., Nojiri H., Cronin L. (2012). Directed assembly of nanoscale Co(ii)-substituted {Co_9_[P_2_W_15_]_3_} and {Co_14_[P_2_W_15_]_4_} polyoxometalates. Chem. Commun..

[3] An H.-Y., Wang E.-B., Xiao D.-R., Li Y.-G., Su Z.-M., Xu L. (2006). Chiral 3D Architectures with Helical Channels Constructed from Polyoxometalate Clusters and Copper–Amino Acid Complexes. Angew. Chem. Int. Ed..

[4] Long D.-L., Tsunashima R., Cronin L. (2010). Polyoxometalates: Building Blocks for Functional Nanoscale Systems. Angew. Chem. Int. Ed..

[5] Oms O., Dolbecq A., Mialane P. (2012). Diversity in structures and properties of 3d-incorporating polyoxotungstates. Chem. Soc. Rev..

[6] Zou C., Zhang Z., Xu X., Gong Q., Li J., Wu C.-D. (2012). A Multifunctional Organic–Inorganic Hybrid Structure Based on Mn^III^–Porphyrin and Polyoxometalate as a Highly Effective Dye Scavenger and Heterogenous Catalyst. J. Am. Chem. Soc..

[7] Lv H., Geletii Y.V., Zhao C., Vickers J.W., Zhu G., Luo Z., Song J., Lian T., Musaev D.G., Hill C.L. (2012). Polyoxometalate water oxidation catalysts and the production of green fuel. Chem. Soc. Rev..

[8] Lv H., Song J., Geletii Y.V., Vickers J.W., Sumliner J.M., Musaev D.G., Kögerler P., Zhuk P.F., Bacsa J., Zhu G. (2014). An Exceptionally Fast Homogeneous Carbon-Free Cobalt-Based Water Oxidation Catalyst. J. Am. Chem. Soc..

[9] Wang S.-S., Kong X.-Y., Wu W., Wu X.-Y., Cai S., Lu C.-Z. (2022). Synergic coordination of multicomponents for the formation of a {Ni_30_} cluster substituted polyoxometalate and its in-situ assembly. Inorg. Chem. Front..

[10] Chen Y., Guo Z.-W., Chen Y.-P., Zhuang Z.-Y., Wang G.-Q., Li X.-X., Zheng S.-T., Yang G.-Y. (2021). Two novel nickel cluster substituted polyoxometalates: Syntheses, structures and their photocatalytic activities, magnetic behaviors, and proton conduction properties. Inorg. Chem. Front..

[11] Liu J.-C., Wang J.-F., Han Q., Shangguan P., Liu L.-L., Chen L.-J., Zhao J.-W., Streb C., Song Y.-F. (2021). Multicomponent Self-Assembly of a Giant Heterometallic Polyoxotungstate Supercluster with Antitumor Activity. Angew. Chem. Int. Ed..

[12] Zhao M., Zhu X.-Y., Li Y.-Z., Chang J.-N., Li M.-X., Ma L.-H., Guo X.-Y. (2022). A Lindqvist-type [W_6_O_19_]^2−^ organic–inorganic compound: Synthesis, characterization, antibacterial activity and preliminary studies on the mechanism of action. Tungsten.

[13] Bijelic A., Aureliano M., Rompel A. (2019). Polyoxometalates as Potential Next-Generation Metallodrugs in the Combat Against Cancer. Angew. Chem. Int. Ed..

[14] Rhule J.T., Hill C.L., Judd D.A., Schinazi R.F. (1998). Polyoxometalates in medicine. Chem. Rev..

[15] Cardona-Serra S., Clemente-Juan J.M., Coronado E., Gaita-Ariño A., Suaud N., Svoboda O., Bastardis R., Guihéry N., Palacios J.J. (2015). Electrically Switchable Magnetic Molecules: Inducing a Magnetic Coupling by Means of an External Electric Field in a Mixed-Valence Polyoxovanadate Cluster. Chem. Eur. J..

[16] Clemente-Juan J.M., Coronado E., Gaita-Ariño A. (2012). Magnetic polyoxometalates: From molecular magnetism to molecular spintronics and quantum computing. Chem. Soc. Rev..

[17] Yan-Qing Jiao C.Q. (2014). Xin-Long Wang, Chun-Gang Wang, Chun-Yi Sun, Hai-Ning Wang, Kui-Zhan Shao, and Zhong-Min Su, Three Cobalt(II)-Linked {P_8_W_48_} Network Assemblies: Syntheses, Structures, and Magnetic and Photocatalysis Properties. Chem. Asian. J..

[18] Ji Y., Huang L., Hu J., Streb C., Song Y.-F. (2015). Polyoxometalate-functionalized nanocarbon materials for energy conversion, energy storage and sensor systems. Energ. Environ. Sci..

[19] Li Q., Zhang L., Dai J., Tang H., Li Q., Xue H., Pang H. (2018). Polyoxometalate-based materials for advanced electrochemical energy conversion and storage. Chem. Eng. J..

[20] Chen L., Chen W.-L., Wang X.-L., Li Y.-G., Su Z.-M., Wang E.-B. (2019). Polyoxometalates in dye-sensitized solar cells. Chem. Soc. Rev..

[21] Koshevar V.D., Shkadretsova V.G., Kazhuro I.P. (2021). Photochemical Engineering and Thermal Transformations of Molybdenum Polyoxometallate Complexes in Aluminosilicate Matrices. Inorg. Mater..

[22] Hiskia A., Mylonas A., Papaconstantinou E. (2001). Comparison of the photoredox properties of polyoxometallates and semiconducting particles. Chem. Soc. Rev..

[23] Liang Z., Zhang D., Ma P., Niu J., Wang J. (2015). A {Co_4_O_4_} Cubane Incorporated within a Polyoxoniobate Cluster. Chem. Eur. J..

[24] Ibrahim M., Haider A., Xiang Y., Bassil B.S., Carey A.M., Rullik L., Jameson G.B., Doungmene F., Mbomekallé I.M., de Oliveira P. (2015). Tetradecanuclear Iron(III)-Oxo Nanoclusters Stabilized by Trilacunary Heteropolyanions. Inorg. Chem..

[25] Duan Y., Clemente-Juan J.M., Giménez-Saiz C., Coronado E. (2016). Cobalt Clusters with Cubane-Type Topologies Based on Trivacant Polyoxometalate Ligands. Inorg. Chem..

[26] Car P.-E., Guttentag M., Baldridge K.K., Alberto R., Patzke G.R. (2012). Synthesis and characterization of open and sandwich-type polyoxometalates reveals visible-light-driven water oxidation via POM-photosensitizer complexes. Green Chem..

[27] Al-Oweini R., Sartorel A., Bassil B.S., Natali M., Berardi S., Scandola F., Kortz U., Bonchio M. (2014). Photocatalytic Water Oxidation by a Mixed-Valent Mn^III^_3_Mn^IV^O_3_ Manganese Oxo Core that Mimics the Natural Oxygen-Evolving Center. Angew. Chem. Int. Ed..

[28] Ibrahim M., Haider A., Lan Y., Bassil B.S., Carey A.M., Liu R., Zhang G., Keita B., Li W., Kostakis G.E. (2014). Multinuclear Cobalt(II)-Containing Heteropolytungstates: Structure, Magnetism, and Electrochemistry. Inorg. Chem..

[29] Geletii Y.V., Huang Z., Hou Y., Musaev D.G., Lian T., Hill C.L. (2009). Homogeneous Light-Driven Water Oxidation Catalyzed by a Tetraruthenium Complex with All Inorganic Ligands. J. Am. Chem. Soc..

[30] Song F., Ding Y., Ma B., Wang C., Wang Q., Du X., Fu S., Song J. (2013). K_7_[Co^III^Co^II^(H_2_O)W_11_O_39_]: A molecular mixed-valence Keggin polyoxometalate catalyst of high stability and efficiency for visible light-driven water oxidation. Energy Environ. Sci..

[31] Du X., Ding Y., Song F., Ma B., Zhao J., Song J. (2015). Efficient photocatalytic water oxidation catalyzed by polyoxometalate [Fe_11_(H_2_O)_14_(OH)_2_(W_3_O_10_)_2_(*α*-SbW_9_O_33_)_6_]^27−^ based on abundant metals. Chem. Commun..

[32] Folkman S.J., Finke R.G. (2017). Electrochemical Water Oxidation Catalysis Beginning with Co(II) Polyoxometalates: The Case of the Precatalyst Co_4_V_2_W_18_O_68_^10–^. ACS Catal..

[33] Shahsavarifar S., Masteri-Farahani M., Ganjali M.R. (2021). New Water Oxidation Electrocatalyst Based on the Cobalt-Containing Polyoxometalate-Reduced Graphene Oxide Hybrid Nanomaterial. Langmuir.

[34] Haider A., Bassil B.S., Soriano-López J., Qasim H.M., Sáenz de Pipaón C., Ibrahim M., Dutta D., Koo Y.-S., Carbó J.J., Poblet J.M. (2019). 9-Cobalt(II)-Containing 27-Tungsto-3-germanate(IV): Synthesis, Structure, Computational Modeling, and Heterogeneous Water Oxidation Catalysis. Inorg. Chem..

[35] Schiwon R., Klingan K., Dau H., Limberg C. (2014). Shining light on integrity of a tetracobalt-polyoxometalate water oxidation catalyst by X-ray spectroscopy before and after catalysis. Chem. Comm..

[36] Li N., Liu J., Dong B.-X., Lan Y.-Q. (2020). Polyoxometalate-Based Compounds for Photo- and Electrocatalytic Applications. Angew. Chem. Int. Ed..

[37] Chen W.C., Wang X.L., Qin C., Shao K.Z., Su Z.M., Wang E.B. (2016). A carbon-free polyoxometalate molecular catalyst with a cobalt-arsenic core for visible light-driven water oxidation. Chem. Commun..

[38] Al-Sayed E., Nandan S.P., Tanuhadi E., Giester G., Arrigoni M., Madsen G.K.H., Cherevan A., Eder D., Rompe A. (2021). Phosphate-Templated Encapsulation of a {Co^II^_4_O_4_} Cubane in Germanotungstates as Carbon-Free Homogeneous Water Oxidation Photocatalysts. ChemSusChem.

[39] Bai D., Zhou C.W., Zhang J.Y., Yuan Y., Geng S.Y., Xie Z.Y., Xia F.W., Shi S.Y., Du L. (2022). Two Novel Catalysts Based on Nickel-Substituted POMs Hybrids for Photocatalytic H_2_ Evolution from Water Splitting. J. Clust. Sci..

[40] Cui T., Qin L., Fu F., Xin X., Li H., Fang X., Lv H. (2021). Pentadecanuclear Fe-Containing Polyoxometalate Catalyst for Visible-Light-Driven Generation of Hydrogen. Inorg. Chem..

[41] Du X., Zhao J., Mi J., Ding Y., Zhou P., Ma B., Zhao J., Song J. (2015). Efficient photocatalytic H_2_ evolution catalyzed by an unprecedented robust molecular semiconductor {Fe_11_} nanocluster without cocatalysts at neutral conditions. Nano Energy.

[42] Feng Y., Qin L., Zhang J., Fu F., Li H., Xiang H., Lv H. (2022). Wheel-shaped icosanuclear Cu-containing polyoxometalate catalyst: Mechanistic and stability studies on light-driven hydrogen generation. Chin. J. Catal..

[43] Sato K., Yonesato K., Yatabe T., Yamaguchi K., Suzuki K. (2022). Nanostructured Manganese Oxides within a Ring-Shaped Polyoxometalate Exhibiting Unusual Oxidation Catalysis. Chem. Eur. J..

[44] Bassil B.S., Al-Oweini M.I.R., Asano M., Wang Z., van Tol J., Dalal N.S., Choi K.-Y., Biboum R.N., Keita B., Nadjo L. (2011). A Planar {Mn_19_(OH)_12_}^26+^ Unit Incorporated in a 60-Tungsto-6-Silicate Polyanion. Angew. Chem. Int. Ed..

[45] Fang X., Kögerler P., Furukawa Y., Speldrich M., Luban M. (2011). Molecular Growth of a Core–Shell Polyoxometalate. Angew. Chem. Int. Ed..

[46] Mal S.S., Dickman M.H., Kortz U., Todea A.M., Merca A., Bögge H., Glaser T., Müller A., Nellutla S., Kaur N. (2008). Nucleation Process in the Cavity of a 48-Tungstophosphate Wheel Resulting in a 16-Metal-Centre Iron Oxide Nanocluster. Chem. Eur. J..

[47] Godin B., Chen Y.-G., Vaissermann J., Ruhlmann L., Verdaguer M., Gouzerh P. (2005). Coordination Chemistry of the Hexavacant Tungstophosphate [H_2_P_2_W_12_O_48_]^12−^ with Fe^III^ Ions: Towards Original Structures of Increasing Size and Complexity. Angew. Chem. Int. Ed..

[48] Goura J., Bassil B.S., Bindra J.K., Rutkowska I.A., Kulesza P.J., Dalal N.S., Kortz U. (2020). Fe^III^_48_-Containing 96-Tungsto-16-Phosphate: Synthesis, Structure, Magnetism and Electrochemistry. Chem. Eur. J..

[49] Goberna-Ferrón S., Vigara L., Soriano-López J., Galán-Mascarós J.R. (2012). Identification of a Nonanuclear {Co^II^_9_} Polyoxometalate Cluster as a Homogeneous Catalyst for Water Oxidation. Inorg. Chem..

[50] Ibrahim M., Lan Y., Bassil B.S., Xiang Y., Suchopar A., Powell A.K., Kortz U. (2011). Hexadecacobalt(II)-containing polyoxometalate-based single-molecule magnet. Angew. Chem. Int. Ed..

[51] Han X.B., Zhang Z.M., Zhang T., Li Y.G., Lin W., You W., Su Z.M., Wang E.B. (2014). Polyoxometalate-based cobalt-phosphate molecular catalysts for visible light-driven water oxidation. J. Am. Chem. Soc..

[52] Shi D., Cui C.-J., Sun C.-X., Du J.-P., Liu C.-S. (2020). A new [Co_21_(H_2_O)_4_(OH)_12_]^30+^ unit-incorporating polyoxotungstate for sensitive detection of dichlorvos. N. J. Chem..

[53] Yao S., Zhang Z., Li Y., Wang E. (2009). A {Cu_6_}-containing inorganic-metal-organic sandwich-type tungstoantimonite and its 3D supramolecular framework. Inorg. Chem. Commun..

[54] Mialane P., Dolbecq A., Marrot J., Rivière E., Sécheresse F. (2003). A Supramolecular Tetradecanuclear Copper(II) Polyoxotungstate. Angew. Chem. Int. Ed..

[55] Mal S.S., Kortz U. (2005). The Wheel-Shaped Cu_20_ Tungstophosphate [Cu_20_Cl(OH)_24_(H_2_O)_12_(P_8_W_48_O_184_)]^25−^ Ion. Angew. Chem. Int. Ed..

[56] Han X.B., Li Y.G., Zhang Z.M., Tan H.Q., Lu Y., Wang E.B. (2015). Polyoxometalate-based nickel clusters as visible light-driven water oxidation catalysts. J. Am. Chem. Soc..

[57] Ibrahim M., Xiang Y., Bassil B.S., Lan Y., Powell A.K., de Oliveira P., Keita B., Kortz U. (2013). Synthesis, Magnetism, and Electrochemistry of the Ni^14-^ and Ni_5_-Containing Heteropolytungstates [Ni_14_(OH)_6_(H_2_O)_10_(HPO_4_)_4_(P_2_W_15_O_56_)_4_]^34–^ and [Ni_5_(OH)_4_(H2O)_4_(*β*-GeW_9_O_34_)(*β*-GeW_8_O_30_(OH))]^13–^. Inorg. Chem..

[58] Han X.-B., Qin C., Wang X.-L., Tan Y.-Z., Zhao X.-J., Wang E.-B. (2017). Bio-inspired assembly of cubane-adjustable polyoxometalate-based high-nuclear nickel clusters for visible light-driven hydrogen evolution. Appl. Catal. B.

[59] Goura J., Bassil B.S., Ma X., Rajan A., Moreno-Pineda E., Schnack J., Ibrahim M., Powell A.K., Ruben M., Wang J. (2021). Ni^II^_36_-Containing 54-Tungsto-6-Silicate: Synthesis, Structure, Magnetic and Electrochemical Studies. Chem. Eur. J..

[60] Yao L., Wei D., Ni Y., Yan D., Hu C. (2016). Surface localization of CdZnS quantum dots onto 2D g-C_3_N_4_ ultrathin microribbons: Highly efficient visible light-induced H_2_-generation. Nano Energy.

[61] Artero V., Fontecave M. (2005). Some general principles for designing electrocatalysts with hydrogenase activity. Coord. Chem. Rev..

[62] Helm M.L., Stewart M.P., Bullock R.M., DuBois M.R., DuBois D.L. (2011). A Synthetic Nickel Electrocatalyst with a Turnover Frequency Above 100,000 s^−1^ for H_2_ Production. Science.

[63] Liu P., Rodriguez J.A. (2005). Catalysts for Hydrogen Evolution from the [NiFe] Hydrogenase to the Ni_2_P(001) Surface: The Importance of Ensemble Effect. J. Am. Chem. Soc..

[64] Lv H., Guo W., Wu K., Chen Z., Bacsa J., Musaev D.G., Geletii Y.V., Lauinger S.M., Lian T., Hill C.L. (2014). A noble-metal-free, tetra-nickel polyoxotungstate catalyst for efficient photocatalytic hydrogen evolution. J. Am. Chem. Soc..

[65] Zhang M., Xin X., Feng Y., Zhang J., Lv H., Yang G.-Y. (2022). Coupling Ni-substituted polyoxometalate catalysts with water-soluble CdSe quantum dots for ultraefficient photogeneration of hydrogen under visible light. Appl. Catal. B.

[66] Chang Q., Meng X., Ruan W., Feng Y., Li R., Zhu J., Ding Y., Lv H., Wang W., Chen G. (2022). Metal–Organic Cages with {SiW_9_Ni_4_} Polyoxotungstate Nodes. Angew. Chem. Int. Ed..

[67] Qin L., Liang F., Li Y., Wu J., Guan S., Wu M., Xie S., Luo M., Ma D. (2022). A 2D Porous Zinc-Organic Framework Platform for Loading of 5-Fluorouracil. Inorganics.

[68] Qin L., Li Y., Liang F., Li L., Lan Y., Li Z., Lu X., Yang M., Ma D. (2022). A microporous 2D cobalt-based MOF with pyridyl sites and open metal sites for selective adsorption of CO_2_. Microporous Mesoporous Mater..

[69] Qin L., Zhao C., Yao L.-Y., Dou H., Zhang M., Xie J., Weng T.-C., Lv H., Yang G.-Y. (2021). Efficient Photogeneration of Hydrogen Boosted by Long-Lived Dye-Modified Ir(III) Photosensitizers and Polyoxometalate Catalyst. CCS Chem..

[70] Herve G., Teze A. (1977). Study of.alpha.- and.beta.-enneatungstosilicates and -germanates. Inorg. Chem..

[71] Zheng M., Cao X., Ding Y., Tian T., Lin J. (2018). Boosting photocatalytic water oxidation achieved by BiVO_4_ coupled with iron-containing polyoxometalate: Analysis the true catalyst. J. Catal..

[72] Pradeep C.P., Long D.-L., Kögerler P., Cronin L. (2007). Controlled assembly and solution observation of a 2.6 nm polyoxometalate ‘super’ tetrahedron cluster: [KFe_12_(OH)_18_(α-1,2,3-P_2_W_15_O_56_)_4_]^29−^. Chem. Commun..

[73] Sheldrick G. (2008). A short history of SHELX. Acta Crystallogr. Sec. A.

[74] Dolomanov O.V., Bourhis L.J., Gildea R.J., Howard J.A.K., Puschmann H. (2009). OLEX2: A complete structure solution, refinement and analysis program. J. Appl. Crystallogr..

[75] Palatinus L., Chapuis G. (2007). SUPERFLIP—A computer program for the solution of crystal structures by charge flipping in arbitrary dimensions. J. Appl. Crystallogr..

[76] Liu Z., Bian Z., Hao F., Nie D., Ding F., Chen Z., Huang C. (2009). Highly efficient, orange–red organic light-emitting diodes using a series of green-emission iridium complexes as hosts. Org. Electron..

[77] Von Allmen K., Moré R., Müller R., Soriano-López J., Linden A., Patzke G.R. (2015). Nickel-Containing Keggin-Type Polyoxometalates as Hydrogen Evolution Catalysts: Photochemical Structure–Activity Relationships. ChemPlusChem.

[78] Guo W., Lv H., Bacsa J., Gao Y., Lee J.S., Hill C.L. (2016). Syntheses, Structural Characterization, and Catalytic Properties of Di- and Trinickel Polyoxometalates. Inorg. Chem..

[79] Paille G., Boulmier A., Bensaid A., Ha-Thi M.-H., Tran T.-T., Pino T., Marrot J., Riviere E., Hendon C.H., Oms O. (2019). An unprecedented {Ni_14_SiW_9_} hybrid polyoxometalate with high photocatalytic hydrogen evolution activity. Chem. Commun..

[80] Lv H., Chi Y., van Leusen J., Koegerler P., Chen Z., Bacsa J., Geletii Y.V., Guo W., Lian T., Hill C.L. (2015). {Ni_4_(OH)_3_AsO_4_}_4_(B-α-PW_9_O_34_)_4_^28-^: A New Polyoxometalate Structural Family with Catalytic Hydrogen Evolution Activity. Chem. Eur. J..

