# Supplementary material for: Structurally-New Hexadecanuclear Ni-Containing Silicotungstate with Catalytic Hydrogen Generation Activity
Source: Molecules. 2023 Feb 21;28(5):2017. doi: 10.3390/molecules28052017 (PMC10004391; doi:10.3390/molecules28052017)
Supplement: Supplementary file 1 [file molecules-28-02017-s001.zip › molecules-2125698-supplementary.pdf]

Structurally-New Hexadecanuclear Ni-Containing Silicotungstate with Catalytic Hydrogen Generation Activity

Yequn Wang, Xing Xin, Yeqin Feng, Manzhou Chi, Ruijie Wang, Tianfu Liu, Hongjin Lv*

MOE Key Laboratory of Cluster Science, Beijing Key Laboratory of Photoelectroic/Electrophotonic Conversion Materials, School of Chemistry and Chemical Engineering, Beijing Institute of Technology, Beijing 102488, P. R. China.

*Correspondence: hlv@bit.edu.cn

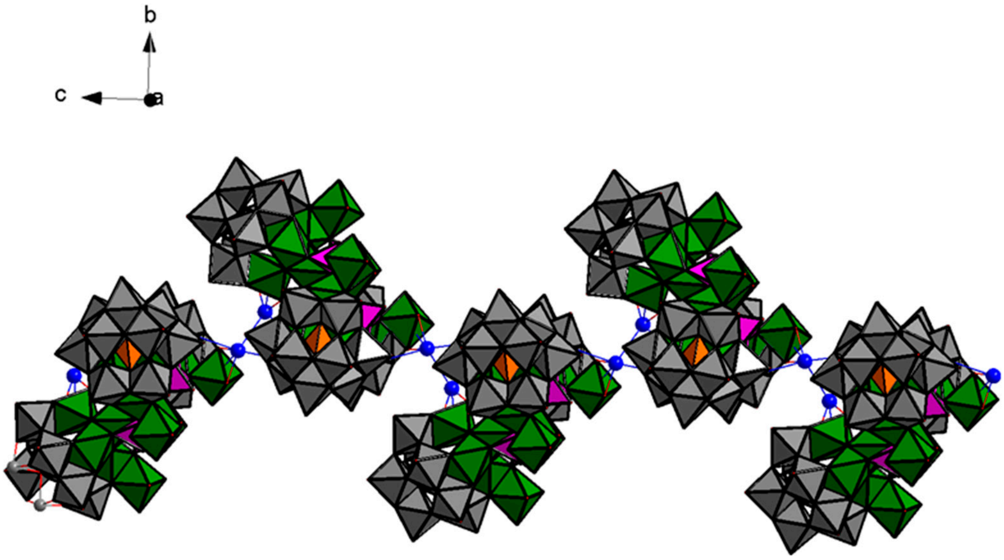

Figure S1. 1-D chain viewed along the *a* axis of the building blocks of polyoxoanion Na-**$Ni_{16}P_4(SiW_9)_3$**. Color code: WO_6 = grey octahedra; PO_4 = pink tetrahedral; SiO_4 = orange tetrahedra; NiO_6 = green octahedron; Na = blue balls.

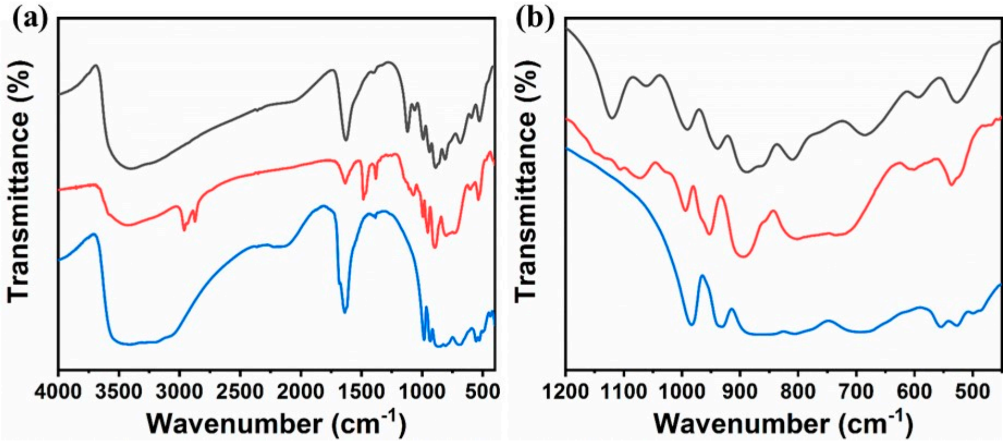

Figure S2. FT-IR spectra of Na^+ salt (black curves) and TBA^+ salt (red curves) of **$Ni_{16}P_4(SiW_9)_3$** polyoxoanions and lacunary $[A\text{-}\alpha\text{-}SiW_9O_{34}]^{10-}$ POM ligand (blue curves) in the range of (a) 4000 to 400 cm^{-1} and (b) 1200 to 400 cm^{-1}, ~ 2 wt % in KBr pellet.

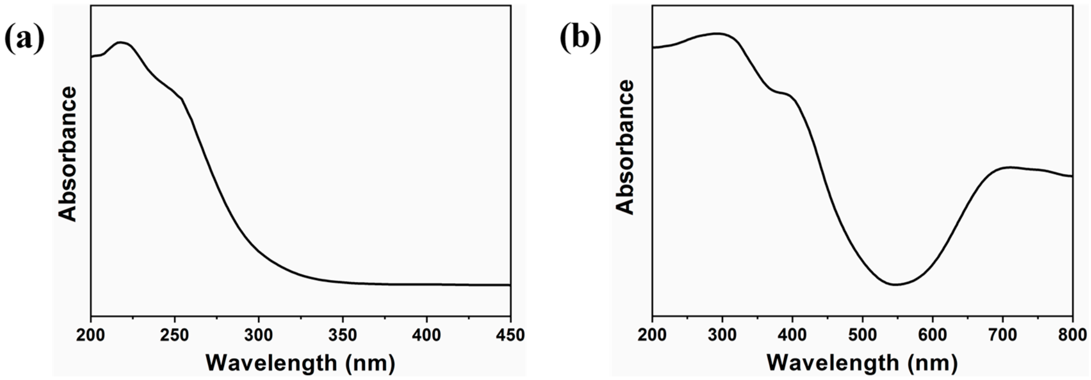

Figure S3. The solution UV-vis adsorption spectra and the solid-state of UV-vis adsorption spectra of Na-$\mathbf{Ni_{16}P_4(SiW_9)_3}$ (a and b).

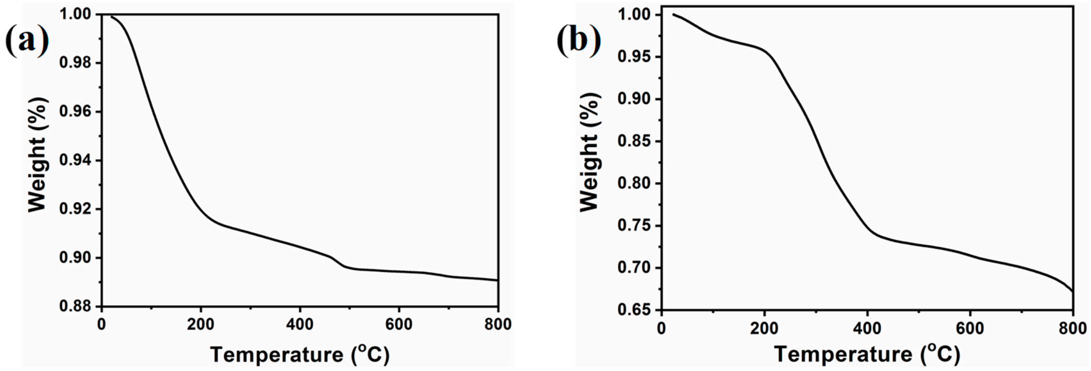

Figure S4. Thermogravimetric analyses of (a) Na-$\mathbf{Ni_{16}P_4(SiW_9)_3}$ and (b) TBA-$\mathbf{Ni_{16}P_4(SiW_9)_3}$.

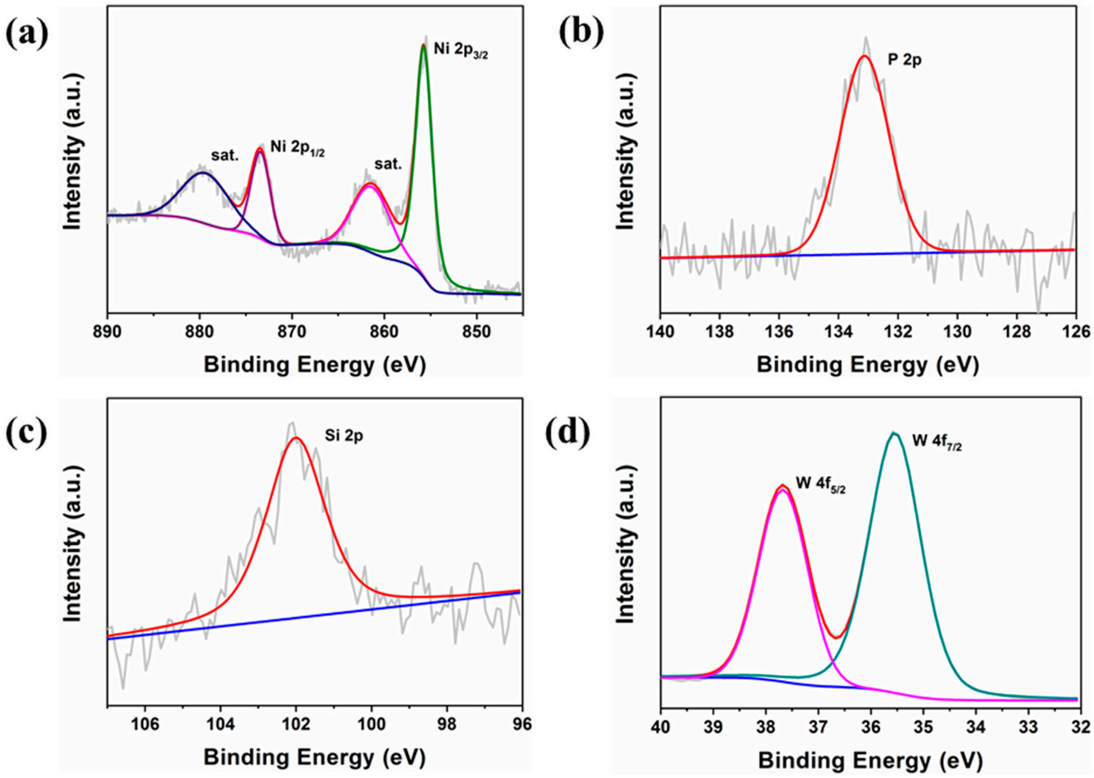

Figure S5. X-ray photoelectron spectroscopy of Ni (a), P (b), Si (c), and W (d) in the Na-**$Ni_{16}P_4(SiW_9)_3$**.

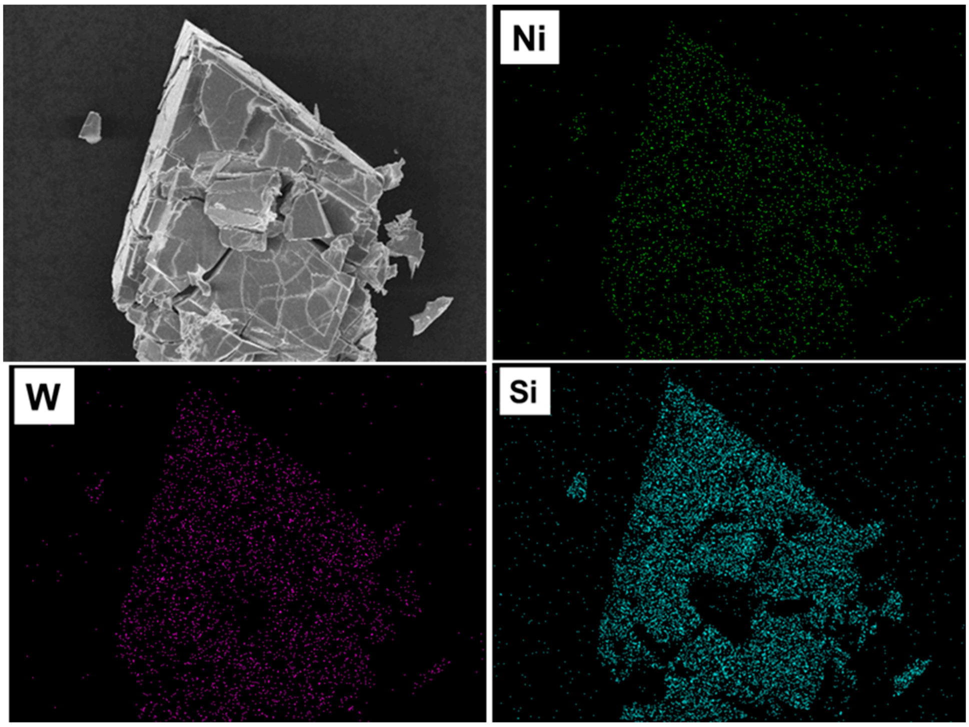

Figure S6. SEM images of the Na-**$Ni_{16}P_4(SiW_9)_3$** sample and the corresponding EDX elemental maps of Ni, W, and Si.

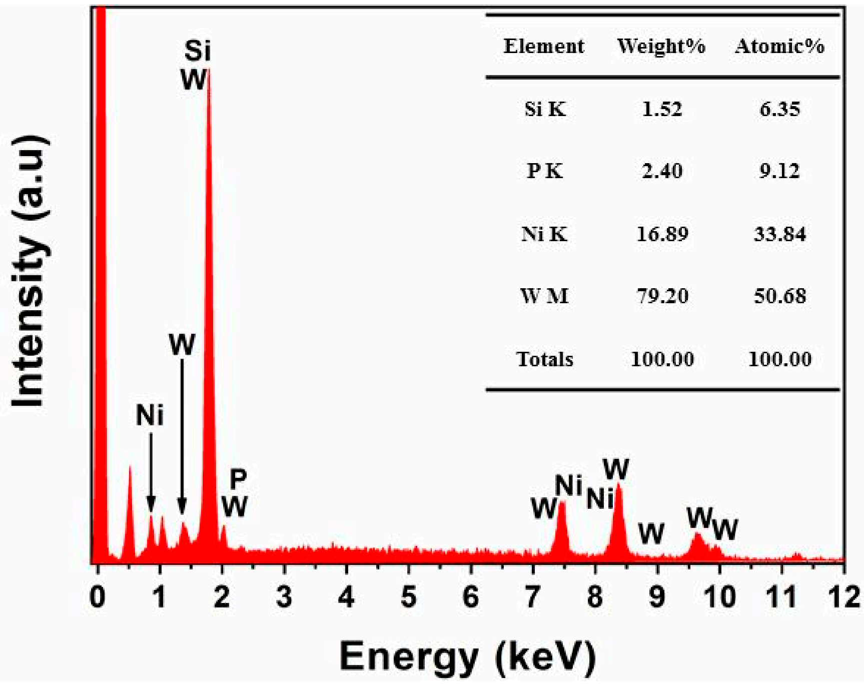

Element	Weight%	Atomic%
Si K	1.52	6.35
P K	2.40	9.12
Ni K	16.89	33.84
W M	79.20	50.68
Totals	100.00	100.00

Figure S7. EDS analysis of the Na-$\mathbf{Ni_{16}P_4(SiW_9)_3}$.

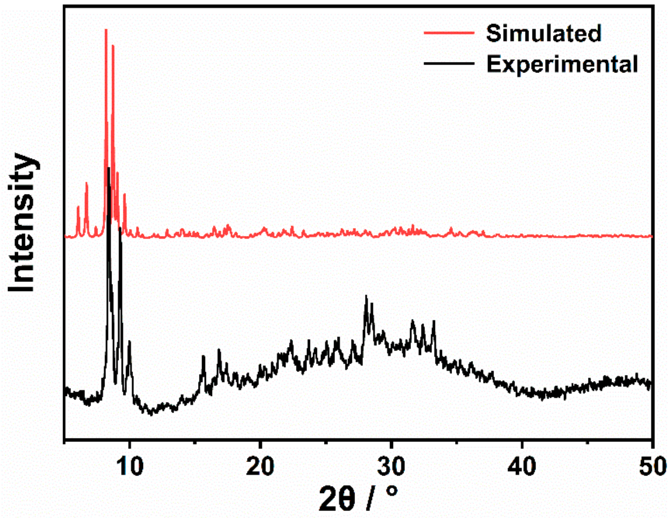

Figure S8. The experimental (black) and simulated (red) PXRD patterns of the Na-$\mathbf{Ni_{16}P_4(SiW_9)_3}$ compound.

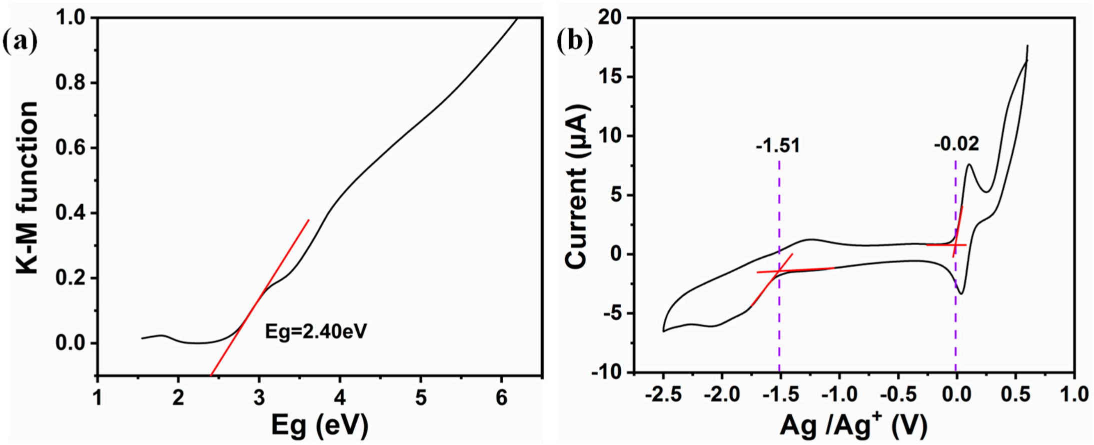

Figure S9. (a) The diffuse reflectance UV-vis-NIR spectra of K-M function vs. energy (eV) and (b) CV curves of TBA-$\mathbf{Ni_{16}P_4(SiW_9)_3}$ at scan rates in 50mV. Condition: 0.1 M tetrabutylammonium hexafluorophosphate ($TBAPF_6$) electrolyte in 10mL of CH_3CN/DMF (v/v = 1/3), deaerated with N_2; glassy carbon working electrode, Pt wire as counter electrode, non-aqueous Ag/Ag^+ as reference electrode (0.1 M $TBAPF_6$ and 0.01 M $AgNO_3$). Ferrocene is used in this experiment as a known reference to calculate the E_{ox} or E_{red}.

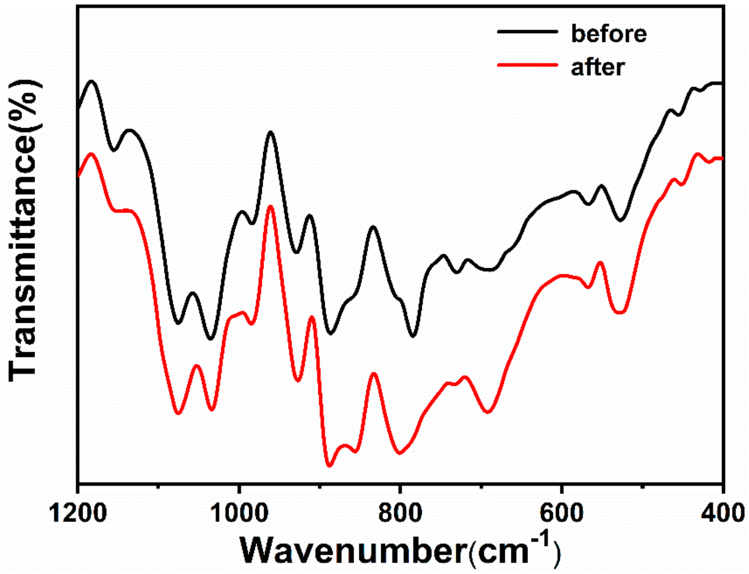

Figure S10. FT-IR spectra of recovered {$[Ru(bpy)_3]_x$-$\mathbf{Ni_{16}P_4(SiW_9)_3}$} before (black) and after (red) photocatalytic H_2 evolution.

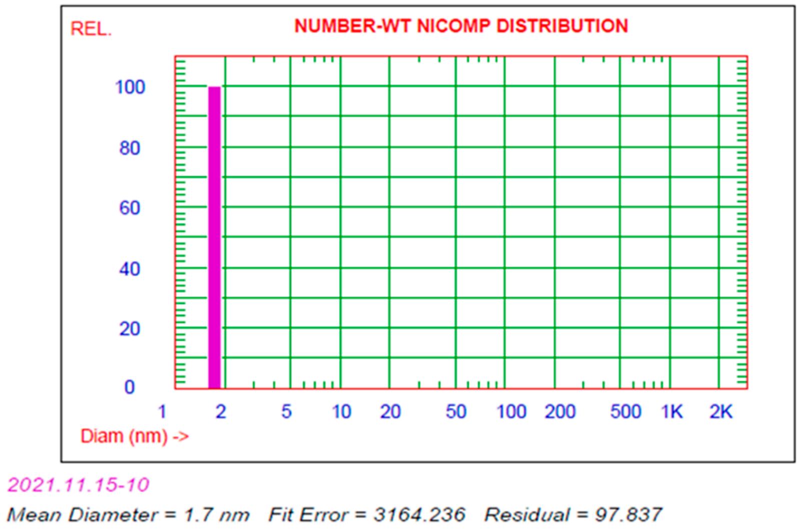

Figure S11. DLS measurement of TBA-**$Ni_{16}P_4(SiW_9)_3$** (20 μM) solution after 6 hours of visible-light irradiation. Conditions: 300W Xe lamp with a 400 nm cutoff filter, $[Ir(coumarin)_2(dtbbpy)]^+$ (0.3 mM), TEOA (0.25 M), TBA-**$Ni_{16}P_4(SiW_9)_3$** (20 μM), CH_3CN/DMF (v/v = 1/3), H_2O (2 M) dearated with Ar/CH_4 (v/v = 4/1).

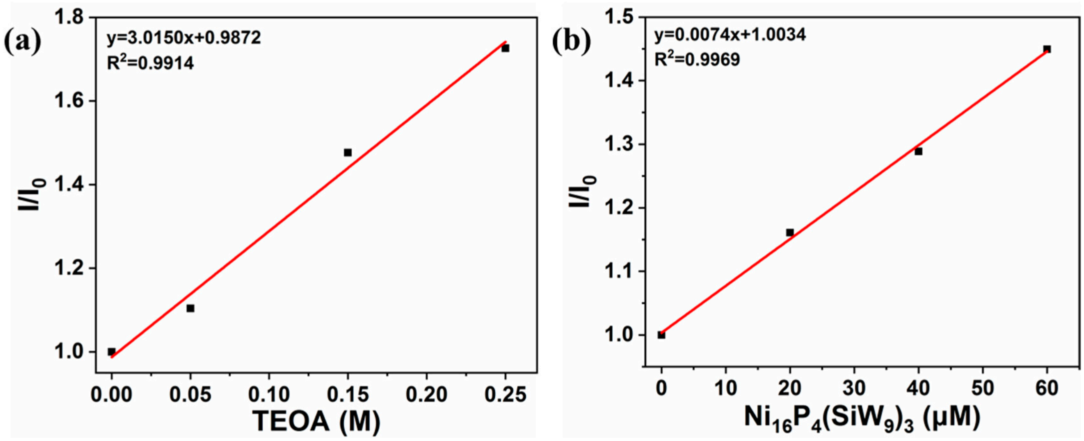

Figure S12. Stern-Volmer plots for emission spectra of $[Ir(coumarin)_2(dtbbpy)][PF_6]$ (0.2 mM) with different concentrations of TBA-**$Ni_{16}P_4(SiW_9)_3$** (0–60 μM) (b) and TEOA (0-0.25 M) (a).

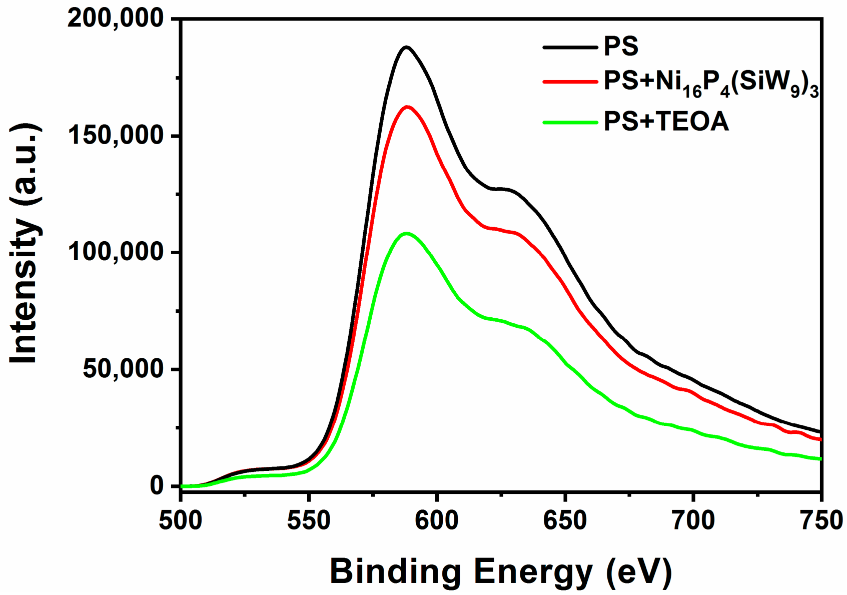

Figure S13. Emission spectra (λ_{ex} = 460 nm) of $[Ir(coumarin)_2(dtbbpy)]^+$ (0.3 mM) (black), with added 20 μM TBA-$\mathbf{Ni_{16}P_4(SiW_9)_3}$ (green) or 0.25M TEOA (blue). Conditions: degassing by Ar.

Table S1. BVS values of Ni, P, Si, W atoms for Na-$\mathbf{Ni_{16}P_4(SiW_9)_3}$.

Atoms	BVS calculation	Atoms	BVS calculation	Atoms	BVS calculation
Ni1	1.88	P2	5.13	W12	5.98
Ni2	1.87	P3	4.83	W13	6.23
Ni3	2.00	P4	5.11	W14	6.02
Ni4	1.89	Si2	4.23	W15	6.09
Ni5	2.04	Si3	3.82	W16	5.99
Ni6	2.14	Si4	3.75	W17	5.79
Ni7	1.99	W1	6.15	W18	6.22
Ni8	1.96	W2	5.73	W19	6.26
Ni9	1.97	W3	6.33	W20	5.74
Ni10	1.91	W4	6.07	W21	5.88
Ni11	1.91	W5	6.14	W22	5.63
Ni12	1.97	W6	5.91	W23	5.97

Ni13	1.93	W7	6.24	W24	6.09
Ni14	1.99	W8	5.82	W25	6.31
Ni15	2.17	W9	6.13	W26	6.07
Ni16	2.00	W10	6.03	W27	5.96
P1	5.01	W11	5.86		

Table S2. BVS values of partial O atoms in Na-$\mathbf{Ni_{16}P_4(SiW_9)_3}$.

O	Bond	Bond length (Å)	BVS calculation	O type
	O(1)-Ni(1)	2.064		
O1	O(1)-Ni(6)	1.994	1.00	OH
	O(1)-Ni(8)	2.096		
	O(4)-Ni(2)	2.076		
O4	O(4)-Ni(3)	1.983	1.12	OH
	O(4)-Ni(9)	2.049		
	O(5)-Ni(4)	2.028		
O5	O(5)-Ni(5)	2.051	1.09	OH
	O(5)-Ni(12)	2.045		
	O(11)-Ni(4)	2.046		
O11	O(11)-Ni(12)	2.020	1.10	OH
	O(11)-Ni(14)	2.088		
	O(15)-Ni(1)	2.056		
O15	O(15)-Ni(6)	2.025	1.07	OH
	O(15)-Ni(16)	2.057		

	O(36)-Ni(4)	2.098		
O36	O(36)-Ni(5)	2.011	1.09	OH
	O(36)-Ni(14)	2.029		
	O(62)-Ni(1)	2.057		
O62	O(62)-Ni(8)	2.047	1.08	OH
	O(62)-Ni(16)	2.052		
	O(82)-Ni(2)	2.079		
O82	O(82)-Ni(9)	1.991	1.09	OH
	O(82)-Ni(11)	2.066		
	O(92)-Ni(2)	2.075		
O92	O(92)-Ni(3)	2.075	1.07	OH
	O(92)-Ni(11)	2.075		
O10	O(10)-Ni(10)	2.040	0.37	H_2O
O26	O(10)-Ni(10)	2.120	0.30	H_2O
O28	O(10)-Ni(7)	2.070	0.34	H_2O
O43	O(10)-Ni(15)	2.030	0.38	H_2O
O57	O(10)-Ni(15)	2.027	0.38	H_2O
O65	O(10)-Ni(7)	2.120	0.30	H_2O
O78	O(10)-Ni(7)	2.000	0.41	H_2O
O83	O(10)-N(i7)	2.180	0.25	H_2O
O106	O(10)-Ni(10)	2.068	0.34	H_2O
O107	O(10)-Ni(13)	2.093	0.32	H_2O
O111	O(10)-Ni(13)	2.081	0.33	H_2O
O122	O(10)-Ni(13)	2.102	0.31	H_2O
O127	O(10)-Ni(13)	2.130	0.29	H_2O
O129	O(10)-Ni(15)	2.125	0.29	H_2O
O131	O(10)-Ni(15)	2.059	0.35	H_2O

Table S3. Comparison of recent literature results for various Ni-POMs catalysts used for the homogeneous photocatalytic hydrogen evolution.

Catalyst	Light Source	Photosensitizer	TON	Time (h)	Ref.
**$\{NiGeW_{11}\}$**		$[Ru(bpy)_3]^{2+}$	36.8	15	[77]
**$\{Ni_3P_2\}$**	LED light	$[Ir(ppy)_2(dtbbpy)]^+$	160	3	[78]
**$\{Ni_{14}P_4(P_2W_{15})_4\}$**	LED light	$[Ir(ppy)_2(dtbbpy)]^+$	260	3.5	[78]
**$\{Ni_4P_2\}$**	LED light	$[Ir(ppy)_2(dtbbpy)]^+$	290	2.5	[64]
**$\{Ni_6B_3(SiW_{10})_2\}$**	Xe lamp	$[Ru(bpy)_3]^{2+}$	83.03	1	[10]
**$\{Ni_{12}P_4(P_2Si_2W_{22})\}$**	Xe lamp	$[Ru(bpy)_3]^{2+}$	23.7	1	[10]
**$[Ni_9P_2(PW_9)_3]$**	Green LED light	CdSe-MPA QDs	10000	12	[65]
**$\{Ni_{14}SiW_9\}$**	Xe lamp	$[Ir(ppy)_2(dtbbpy)]^+$	256	4	[79]
**$[Ni_{16}P_4(AsW_9)_4]$**	LED light	$[Ir(ppy)_2(dtbbpy)]^+$	360	5	[80]
**$\{[Ni_{16}(A\text{-}PW_9)_4]\}$**	LED light	$[Ir(ppy)_2(dtbbpy)]^+$	578.8	12	[58]
**$\{[Ni_{16}(A\text{-}PW_9)_2(B\text{-}PW_9)_2]\}$**	LED light	$[Ir(ppy)_2(dtbbpy)]^+$	679.1	12	[58]
**$\{[Ni_{16}(B\text{-}PW_9)_4]\}$**	LED light	$[Ir(ppy)_2(dtbbpy)]^+$	931.1	12	[58]
**TBA-$Ni_{16}P_4(SiW_9)_3$**	Xe lamp	$[Ir(coumarin)_2(dtbbpy)]^+$	842	6	This work
